# Accuracy, patient-perceived usability, and acceptance of two symptom checkers (Ada and Rheport) in rheumatology: interim results from a randomized controlled crossover trial

**DOI:** 10.1186/s13075-021-02498-8

**Published:** 2021-04-13

**Authors:** Johannes Knitza, Jacob Mohn, Christina Bergmann, Eleni Kampylafka, Melanie Hagen, Daniela Bohr, Harriet Morf, Elizabeth Araujo, Matthias Englbrecht, David Simon, Arnd Kleyer, Timo Meinderink, Wolfgang Vorbrüggen, Cay Benedikt von der Decken, Stefan Kleinert, Andreas Ramming, Jörg H. W. Distler, Nicolas Vuillerme, Achim Fricker, Peter Bartz-Bazzanella, Georg Schett, Axel J. Hueber, Martin Welcker

**Affiliations:** 1 Department of Internal Medicine 3, Friedrich-Alexander-University Erlangen-Nürnberg (FAU) and Universitätsklinikum Erlangen, Ulmenweg 18, 91054 Erlangen, Germany; 2 Deutsches Zentrum für Immuntherapie (DZI), Friedrich-Alexander-University Erlangen-Nürnberg and Universitätsklinikum Erlangen, Erlangen, Germany; 3 Université Grenoble Alpes, AGEIS, Grenoble, France; 4 Verein zur Förderung der Rheumatologie e.V., Würselen, Germany; 5 RheumaDatenRhePort (rhadar), Planegg, Germany; 6 Medizinisches Versorgungszentrum Stolberg, Stolberg, Germany; 7 Klinik für Internistische Rheumatologie, Rhein-Maas Klinikum, Würselen, Germany; 8 Rheumatologische Schwerpunktpraxis, Drs. Kleinert, Rapp, Ronneberger, Schuch U. Wendler, Rheumatology, Erlangen, Germany; 9 Institut Universitaire de France, Paris, France; 10 LabCom Telecom4Health, University of Grenoble Alpes & Orange Labs, Grenoble, France; 11 Qinum GmbH, Cologne, Germany; 12 Section Rheumatology, Sozialstiftung Bamberg, Bamberg, Germany; 13 MVZ für Rheumatologie Dr. Martin Welcker GmbH, Planegg, Germany

**Keywords:** Symptom checker, Diagnosis, eHealth, Accuracy, Apps, Usability, Acceptability, Rheumatology

## Abstract

**Background:**

Timely diagnosis and treatment are essential in the effective management of inflammatory rheumatic diseases (IRDs). Symptom checkers (SCs) promise to accelerate diagnosis, reduce misdiagnoses, and guide patients more effectively through the health care system. Although SCs are increasingly used, there exists little supporting evidence.

**Objective:**

To assess the diagnostic accuracy, patient-perceived usability, and acceptance of two SCs: (1) Ada and (2) Rheport.

**Methods:**

Patients newly presenting to a German secondary rheumatology outpatient clinic were randomly assigned in a 1:1 ratio to complete Ada or Rheport and consecutively the respective other SCs in a prospective non-blinded controlled randomized crossover trial. The primary outcome was the accuracy of the SCs regarding the diagnosis of an IRD compared to the physicians’ diagnosis as the gold standard. The secondary outcomes were patient-perceived usability, acceptance, and time to complete the SC.

**Results:**

In this interim analysis, the first 164 patients who completed the study were analyzed. 32.9% (54/164) of the study subjects were diagnosed with an IRD. Rheport showed a sensitivity of 53.7% and a specificity of 51.8% for IRDs. Ada’s top 1 (D1) and top 5 disease suggestions (D5) showed a sensitivity of 42.6% and 53.7% and a specificity of 63.6% and 54.5% concerning IRDs, respectively. The correct diagnosis of the IRD patients was within the Ada D1 and D5 suggestions in 16.7% (9/54) and 25.9% (14/54), respectively. The median System Usability Scale (SUS) score of Ada and Rheport was 75.0/100 and 77.5/100, respectively. The median completion time for both Ada and Rheport was 7.0 and 8.5 min, respectively. Sixty-four percent and 67.1% would recommend using Ada and Rheport to friends and other patients, respectively.

**Conclusions:**

While SCs are well accepted among patients, their diagnostic accuracy is limited to date.

**Trial registration:**

DRKS.de, DRKS00017642. Registered on 23 July 2019

## Introduction

The European League Again Rheumatism (EULAR) recommendations support that patients with arthritis should be seen as early as possible, ideally during 6 weeks after symptom onset [1], since an early start of the treatment significantly improves patient outcomes [2]. Various strategies have been identified [3, 4] to implement these recommendations; however, the diagnostic delay seems to increase despite such strategies [5, 6].

Symptom checkers (SCs) could improve this situation. SCs are patient-centered diagnostic decision support systems (DDSS) that are designed to offer a scalable, objective, cost-effective, personalized triage strategy. Based on such a triage strategy, SCs should help to receive a more appropriate appointment, for the right patient, at the right time, thus empowering patients. It is known that patients with rheumatic and musculoskeletal diseases (RMD) are highly motivated to use SCs and other medical apps [7]. Thus, SCs like the artificial intelligence-driven Ada have been used to complete more than 15 million health assessments in 130 countries [8].

To ensure the safety and efficacy of such apps, EULAR recently published guidelines [9] that state “self-management apps should be up to date, scientifically justifiable, user-acceptable, and evidence-based where applicable,” and validation should include people with RMDs.

Therefore, the aim of this study was to create real-world-based evidence by evaluating the diagnostic accuracy, usability, acceptance, and completion time of two free, publicly available SCs, Ada (www.ada.com) and Rheport (www.rheport.de).

## Methods

### Study design

We present interim results of a randomized controlled crossover multicenter study, conducted at three centers in Germany. The study was approved by the ethics committee of the Medical Faculty of the University of Erlangen-Nürnberg, Germany (106_19 Bc), reported to the German Clinical Trials Register (DRKS) (DRKS00017642) and conducted in compliance with the Declaration of Helsinki. All patients provided written informed consent before participating. Patients were randomized 1:1 to group 1 (completing Ada first, continuing with Rheport) or group 2 (completing Rheport first, continuing with Ada) by computer-generated block randomization whereas each block contains *n* = 100 patients. SCs were completed before the regular appointment. Assisting personnel was present to help with SC completion if necessary.

### Study patients

Adult patients newly presenting to the first (University Hospital Erlangen, Germany) of three recruiting rheumatology outpatient clinics with musculoskeletal symptoms and unknown diagnosis were included in this cross-sectional study. Patients with a known diagnosis and patients unwilling or unable to comply with the protocol were excluded from the study. Besides the app-related data outlined below, demographic variables, symptom duration, swollen and tender joint count, DAS28 score, ESR, CRP, anti-CCP antibody and rheumatoid factor status, and clinical diagnosis using established classification criteria were recorded. This interim analysis is based on patient data from rheumatology outpatient clinics recorded starting in September 2019 up to February 2020.

### Description of the symptom checkers

**Fig. 1:**
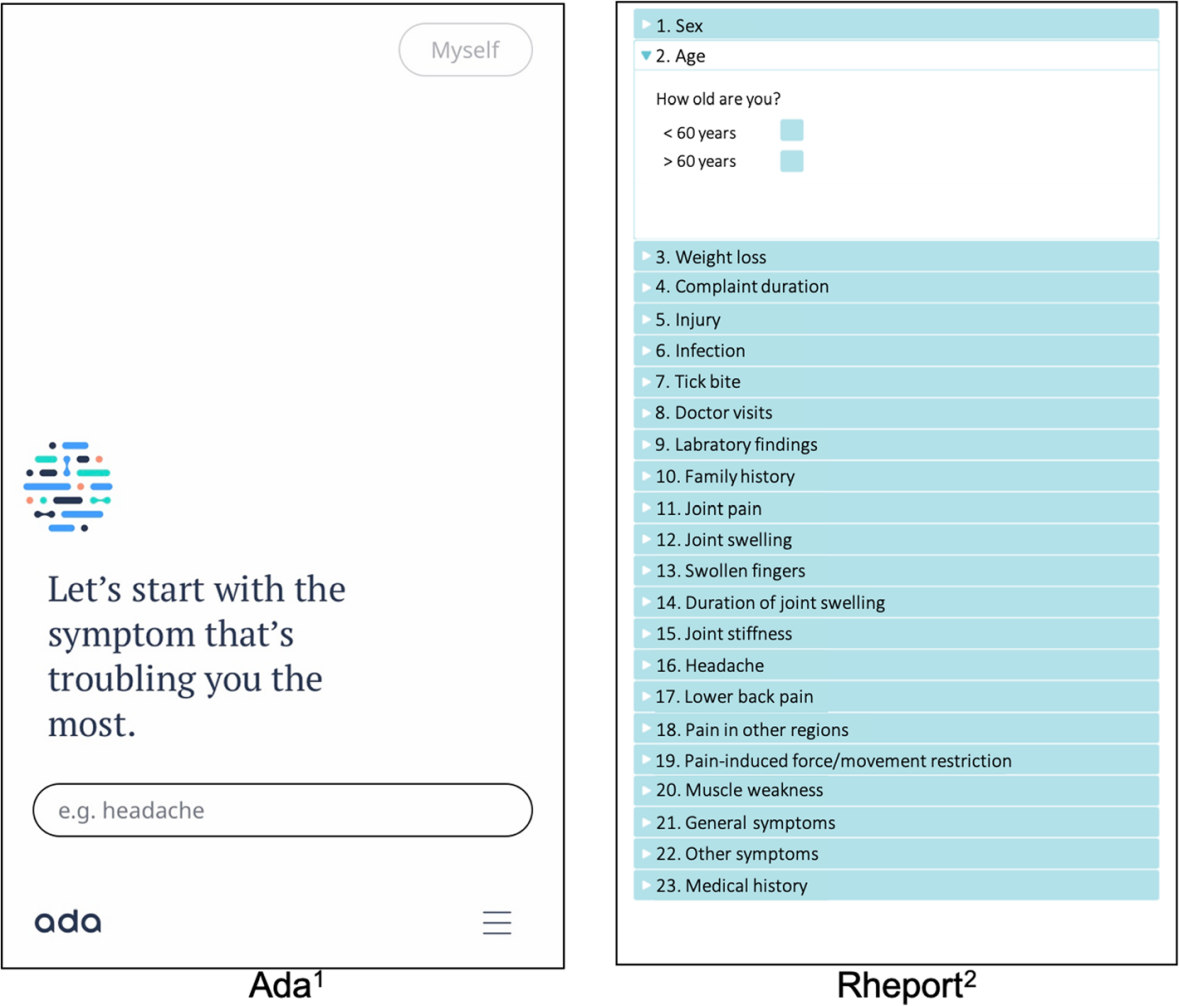
Screenshots of the Ada and Rheport symptom checker. ^1^The German version of Ada was used in the study. ^2^The Rheport menu was translated into English for this figure

Ada is a Conformité Européenne (CE)-certified medical app that is freely available in multiple languages and was used to complete more than 15 million health assessments in 130 countries [8]. The artificial intelligence-driven chatbot app first asks for basic health information (e.g., sex, smoking status) and then asks for the current leading symptoms. The questions (Fig. 1) are dynamically chosen, and the total number varies depending on the previous answers given. Ada then provides a top (D1) and up to 5 concrete disease suggestions (D5), their probability and urgency advice. The app is based on constantly updated research findings and is not limited to RMDs.

Rheport is a rheumatology-specific online platform that uses a fixed patient questionnaire (Fig. 1) including basic health information and rheumatology-specific questions, developed by rheumatologists. A background algorithm calculates the probability of an IRD based on a weighted sum score of the questionnaire answers. A sum score ≥ 1.0 was determined to be the threshold for an IRD. The system is already used in clinical routine to triage appointments of new patients per IRD probability. About 3000 appointments have been organized to date [4]. For this study, an app-based version of the software has been used. Both SCs were tested using three iOS-based tablets.

### Primary outcome

The primary outcome was the diagnostic accuracy regarding the sensitivity and specificity of Ada and Rheport concerning the diagnosis of IRD. The results of the SCs were recorded and compared to the gold standard, i.e., the final physicians’ diagnosis; reported on the discharge summary report; and adjudicated by the head of the local rheumatology department.

### Secondary outcomes

SC completion time and patient-perceived usability were secondary outcomes of this study. SC completion time was measured by supervising local study personnel. Patients completed a survey evaluating the SC usability using the System Usability Scale (SUS) [10]. It consists of 10 statements with 5-point Likert scales ranging from strongly agree to strongly disagree, resulting in a maximum score of 100. Finally, patients were asked if they would recommend the two SCs to friends and other patients.

### Statistical analysis

We performed an interim analysis of the first 164 patients who completed the study. The analysis consisted of (i) a descriptive sample characterization stratified by randomization arm, (ii) an assessment of Ada’s and Rheport’s diagnostic accuracy, and (iii) a descriptive evaluation of the secondary outcome measures specified above for the total sample. Descriptive characteristics for each randomization arm are presented as median (Mdn) and interquartile range (IQR) for interval data and as absolute (*n*) and relative frequency (percent) for nominal data. Comparability of demographic and IRD-related characteristics between the randomization groups was assessed by the Wilcoxon rank-sum tests and *χ*^2^ tests. Diagnostic accuracy was evaluated referring to sensitivity, specificity, negative predictive value (NPV), positive predictive value (PPV), and overall accuracy. The comparability of the secondary outcomes was evaluated by the Wilcoxon signed-rank tests whereas descriptive information is presented as Mdn (IQR). The significance level for inferential tests was set at *p* ≤ 0.05. The software used for the statistical analysis was R (version 3.6.3) and RStudio (version 1.2.5033), respectively.

### Sample size determination

A minimum sample size of *n* = 122 patients was calculated, based on the following assumptions: (1) prevalence, defined as the proportion of subjects who, after presenting to the rheumatologist, are diagnosed with an inflammatory rheumatic disease of 40% [11]; (2) average diagnostic accuracy of previous applications for diagnosis using the 3 most likely diagnoses of 50% [12]; (3) desired accuracy of diagnosis using Ada or Rheport in terms of sensitivity and specificity of 70%; (4) type 1 error: discrete value according to Bujang and Adnan [13] of 4.4%; (5) type 2 error: discrete value according to Bujang and Adnan [13] of 19% and test strength (power) corresponding to 81%.

## Results

### Participants

**Fig. 2:**
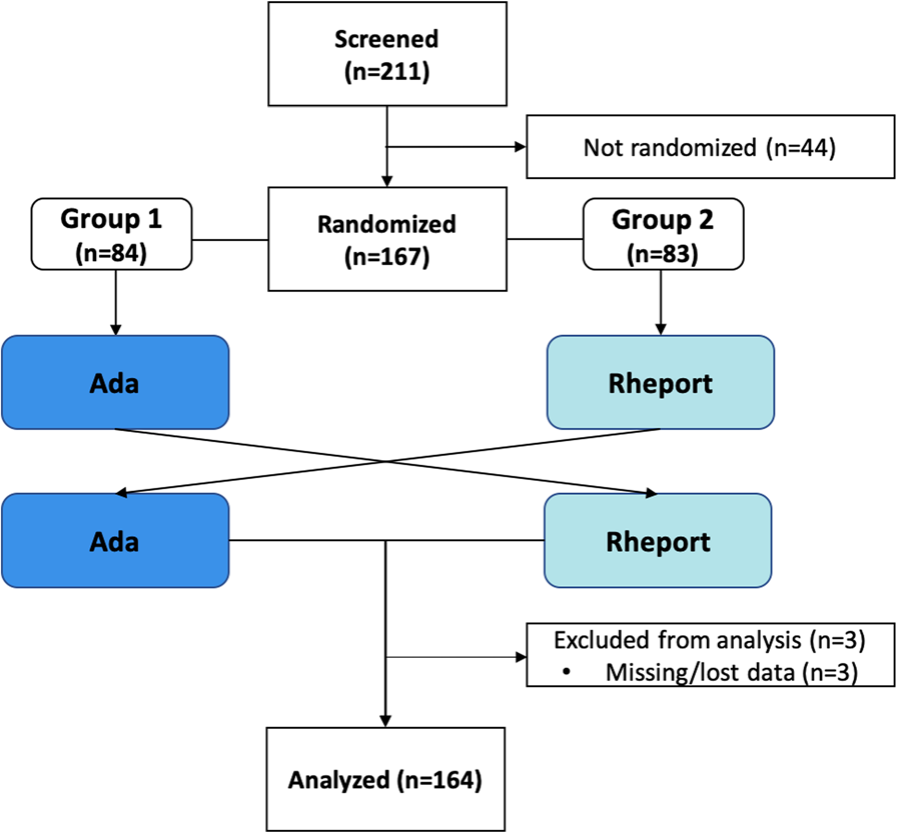
Patient flow diagram

A total of 211 consecutive patients were approached, 167 agreed to participate, and 164 patients were included in the interim analysis presented (Fig. 2). 32.9% (54/164) of the presenting patients were diagnosed with an IRD based on the physicians’ judgment. The classified diagnosis and demographic characteristics are summarized in Tables 1 and 2, respectively.

**Table 1:** Diagnostic categories

**Inflammatory**	
Psoriatic arthritis, % (*N*)	7.9 (13)
Axial spondyloarthritis, % (*N*)	4.9 (8)
Connective tissue disease, % (*N*)	4.9 (8)
Undifferentiated arthritis, % (*N*)	3.7 (6)
Rheumatoid arthritis, % (*N*)	3.0 (5)
Vasculitis, % (*N*)	2.4 (4)
Peripheral spondyloarthritis, % (*N*)	1.2 (2)
Crystal arthropathies, % (*N*)	1.2 (2)
Polymyalgia rheumatica, % (*N*)	1.8 (3)
Other inflammatory RMDs, % (*N*)	1.8 (3)
**Non-inflammatory**	
Other non-inflammatory, % (*N*)	48.2 (79)
Osteoarthritis, % (*N*)	15.2 (25)
Fibromyalgia, % (*N*)	3.7 (6)

**Table 2:** Demographic and health characteristics

	Total sample			
	*N*	Mdn	IQR 25%	IQR 75%
Age (years)	164	51.0	38.8	61.0
Tender joint count 28 (*N*)	163	0.0	0.0	1.0
Swollen joint count 28 (*N*)	163	0.0	0.0	0.0
VAS patient global (mm)	164	50.0	30.0	62.5
ESR (mm/h)	144	9.0	5.0	18.0
CRP (mg/L)	159	5.0	5.0	6.0
DAS28 (CRP)	143	2.4	1.7	3.1
	*n*		%	
Female	113		68.9	
RF (positive)	23/153		15.0	
ACPA (positive)	7/142		4.9	
	IRD patients			
	*N*	Mdn	IQR 25%	IQR 75%
Age (years)	54	54.0	38.2	67.8
Tender joint count 28 (*N*)	53	0.0	0.0	2.0
Swollen joint count 28 (*N*)	53	0.0	0.0	1.0
VAS patient global (mm)	54	30.0	70.0	62.5
ESR (mm/h)	43	17.0	6.5	38.0
CRP (mg/L)	51	5.0	5.0	11.0
DAS28 (CRP)	42	3.1	2.4	4.3
	*n*		%	
Female	30/54		55.6	
RF (positive)	12/50		24.0	
ACPA (positive)	4/50		8.0	
	Non-IRD patients			
	*N*	Mdn	IQR 25%	IQR 75%
Age (years)	110	50.0	39.0	58.0
Tender joint count 28 (*N*)	110	0.0	0.0	0.0
Swollen joint count 28 (*N*)	110	0.0	0.0	0.0
VAS patient global (mm)	110	50.0	20.0	60.0
ESR (mm/h)	101	8.0	4.0	12.0
CRP (mg/L)	108	5.0	5.0	5.0
DAS28 (CRP)	101	2.2	1.7	2.7
	*n*		%	
Female	83/110		75.5	
RF (positive)	11/103		10.7	
ACPA (positive)	3 / 92		3.3	

*Annotation*: *Mdn*, median; *IQR 25%*, interquartile range (25% bound); *IQR 75%*, interquartile range (75% bound)

### Primary outcome

Rheport showed a sensitivity of 53.7% (29/54) and a specificity of 51.8% (57/110). Ada’s D1 and D5 suggestions showed a sensitivity of 42.6% (23/54) and 53.7% (29/54) and a specificity of 63.6% (70/110) and 54.5% (60/110) concerning IRD, respectively. The diagnostic accuracy in the two randomization arms seemed to be similar regarding the individual characteristics of diagnostic accuracy. Further details on the SCs’ diagnostic accuracy can be taken from Table 3. The correct diagnosis of the IRD patients was within Ada’s D1 and D5 suggestions in 16.7% (9/54) and 25.9% (14/54), respectively. The 14 correct ADA D5 disease suggestions encompassed the following diagnosis: 5 PsA, 4 SpA, 3 RA, 2 PMR, and one connective tissue disease (systemic sclerosis) cases.

**Table 3:** Sensitivity, specificity, PPV, NPV, and overall accuracy of Ada and Rheport for the diagnosis of inflammatory rheumatic diseases including 95% confidence intervals

	Characteristic	Symptom checker		
		Rheport	Ada D1	Ada D5
**Total sample** (*n* = 164)	Sensitivity (%)	53.7 (39.7–67.2)	42.6 (29.5–56.7)	53.7 (39.7–67.2)
	Specificity (%)	51.8 (42.1–61.4)	63.6 (53.9–72.4)	54.5 (44.8–64.0)
	PPV (%)	35.4 (25.3–46.8)	36.5 (25.0–49.6)	36.7 (26.4–48.4)
	NPV (%)	69.5 (58.2–78.9)	69.3 (59.2–77.9)	70.6 (59.6–79.7)
	Accuracy (%)	52.4 (44.5–60.2)	56.7 (48.8–64.3)	54.3 (46.3–62.0)
**Ada first** (*n* = 164)	Sensitivity (%)	56.0 (35.3–75.0)	40.0 (21.8–61.1)	60.0 (38.9–78.2)
	Specificity (%)	52.6 (39.1–65.8)	61.4 (47.6–73.7)	54.4 (40.8–67.4)
	PPV (%)	34.1 (20.6–50.7)	31.2 (16.7–50.1)	36.6 (22.6–53.1)
	NPV (%)	73.2 (56.8–85.2)	70.0 (55.2–81.7)	75.6 (59.4–87.1)
	Accuracy (%)	53.7 (42.4–64.6)	54.9 (43.5–65.8)	56.1 (44.7–66.9)
**Rheport first** (*n* = 164)	Sensitivity (%)	51.7 (32.9–70.1)	44.8 (27.0–64.0)	48.3 (29.9–67.1)
	Specificity (%)	50.9 (37.0–64.7)	66.0 (51.6–78.1)	54.7 (40.6–68.2)
	PPV (%)	36.6 (22.6–53.1)	41.9 (25.1–60.7)	36.8 (22.3–54.0)
	NPV (%)	65.9 (49.3–79.4)	68.6 (54.0–80.5)	65.9 (50.0–79.1)
	Accuracy (%)	51.2 (40.0–62.3)	58.5 (47.1–69.1)	52.4 (41.2–63.5)

*Ada D1*, using Ada top suggestion only; *Ada D5*, using all suggestions provided by Ada; *PPV*, positive predictive value; *NPV*, negative predictive value

### Secondary outcomes

The median completion time for Ada and Rheport was 7.0 min (IQR 5.8–9.0) and 8.5 (IQR 8.0–10.0), respectively. On a scale of 0 (worst) to 100 (best), the median SUS of Ada and Rheport was 75.0 (IQR 62.5–85.0) and 77.5 (IQR 62.5–87.5), respectively. Completion time and usability (SUS scores) were not different between the two groups. Sixty-four percent and 67.1% would recommend using Ada and Rheport to friends and other patients, respectively.

## Discussion

This prospective real-world study highlights the currently limited diagnostic accuracy of SCs, such as Ada and Rheport with respect to IRDs. Their overall sensitivity and specificity for IRDs are moderate. SCs offer patients on-demand medical support independent of time and place. An automated SC-based triage, as offered by Rheport, may allow objective, scalable, and transparent decisions. By automating triage decisions, SCs could additionally save money [12, 14] and accelerate the time to correct diagnosis [15], however may also lead to over-diagnosis and over-treatment [16].

Despite increasing patient usage [8], evidence supporting SC effectiveness is limited to date [12, 17]. The results of this study are in line with previous SC analyses [12, 17, 18]. Research supported by Ada Health GmbH shows that Ada had the highest top 3 suggestion diagnostic accuracy (70.5%) compared to other SCs [19], and the correct condition was among the first three results in 83% in an Australian assessment study [20]. Similarly to our results, the majority of patients would recommend Ada (85.3%) to friends or relatives [21].

The first rheumatology-specific SC study with 34 patients [18] showed that only 4 out of 21 patients with inflammatory arthritis were given the first diagnosis of RA or PsA. Proft et al. recently showed that a physician-based referral strategy was more effective than an online self-referral tool for early recognition of axial spondyloarthritis [22]. Nevertheless, these authors recommend using online self-referral tools in addition to traditional referral strategies, as the proportion of axial spondyloarthritis among self-referred patients (19.4%) was clearly higher than the assumed 5% prevalence in patients with chronic back pain. Regarding the current referral sensitivity of 32.9%, complementary SC integration might indeed be part of modern rheumatology.

The diagnostic accuracy of rheumatologists is high based on the comprehensive use of information from patients’ history, symptoms, and also data from laboratory tests and imaging [23]. Therefore, the current comparison of the physicians’ final diagnosis and SC-suggested diagnosis should be interpreted carefully, as the SC diagnosis is based on substantially less data. Furthermore, patients could discuss SC results with their rheumatologists, possibly influencing the rheumatologist’s diagnosis. The sequential usage of both SCs represents a possible bias, as patients might be influenced by the usage of the first SC. However, we could not observe any significant differences related to SC order. The slightly better performance of Ada should be interpreted carefully. In contrast to Rheport, Ada is supported by artificial intelligence and does not use a fixed questionnaire. Ada covers a great variety of different conditions [19] and is not limited to IRDs, whereas Rheport is exclusively meant for the triage of new suspected IRD patients. The study setting was deliberately chosen risk-adverse, so the use of the SCs did not have any clinical implications. Symptom checkers are however designed to be used in community settings, where the probability that a patient will have an IRD is much lower than in a rheumatology clinic and no help for SC completion is available. Furthermore, the exact SC diagnosis might be less important than the SC advice on when to see a doctor, especially in emergency situations. Our study setting caused a much higher a priori chance of having an IRD, as patients were already “screened” by referring physicians. The high proportion of PsA and AxSpA patients is likely attributed to a strong local cooperation with the orthopedic and dermatology department. Additional data from the other two centers will hopefully contribute to balancing results. We did not measure how often help from assisting personnel was necessary for SC completion.

To the best of our knowledge, this is the first prospective, real-world, multicenter study evaluating two currently used SCs in rheumatology. Our results may provide some help to guide and inform patients, treating health care professionals (HCPs) but also other stakeholders in health care. In conclusion, while SCs are well-accepted by patients their diagnostic accuracy is limited. Constant improvement of algorithms might foster the future potential of SCs to improve patient care.

## Data Availability

Data are available on reasonable request from the corresponding author on reasonable request.

## Abbreviations

DDSS: Diagnostic decision support system
EULAR: European League Again Rheumatism
IRD: Inflammatory rheumatic disease
RMD: Rheumatic and musculoskeletal diseases
SC: Symptom checker
SUS: System Usability Scale
